# Estimating the under-five malaria risk in Uganda based on the nearest neighbour matched analysis technique

**DOI:** 10.4314/ahs.v24i2.20

**Published:** 2024-06

**Authors:** Charles Natuhamya

**Affiliations:** Makerere University, School of Public Health

**Keywords:** Indoor residual spraying, insecticide treated nets, long lasting insecticide nets, Malaria, nearest neighbour matching, treatment effects, Uganda

## Abstract

**Introduction:**

Malaria still remains a global burden especially in the under-five despite efforts made towards reducing it. The most recommended vector control methods are; use of insecticide treated nets (ITNs) or long lasting insecticide nets (LLINs) and use of indoor residual spraying (IRS). However, these innovations may not have the same effect on malaria risk in the under-five. This study therefore aimed at assessing; the effect of ITNs/LLINs on malaria risk, the effect of IRS on malaria risk, and the effect of ITNs/LLINs on IRS, using nearest neighbours matched analysis.

**Methods:**

Nearest neighbour matched analysis was used to match the treated and control units by taking each treated unit and searching for the control unit with the nearest neighbours without replacement.

**Results:**

The results revealed a significant and negative effect of ITNs/LLINs and IRS on malaria risk [ATET=-0.05; 95% CI= -0.07 – -0.02] and [ATET=-0.12; 95% CI= -0.15 – -0.09] respectively. It also found a significant and positive effect of ITNs/LLINs on IRS [ATET=0.03; 95% CI= 0.01 – 0.05].

**Conclusions:**

The implementation of policies and programs towards effective use of ITN/LLIN and IRS can reduce the burden of under-five malaria in Uganda.

## Introduction

Malaria is an infection caused by a parasite of the genus Plasmodium^1^ that is transmitted by female anopheles mosquitoes^2^. Despite efforts toward reducing malaria, it remains a global burden 3. In 2018, about 228 million cases of malaria occurred worldwide, of which more than 50% were accounted for by six countries, including Uganda^4^. Sub-Saharan Africa accounted for the majority (95%) of these cases^5^. Globally, children aged under five years are the most vulnerable age group affected by malaria. In 2018, they accounted for 67% (272 000) of all malaria deaths worldwide^4^. In Uganda, approximately 1 in 10 children (9%) aged 0-59 months tested positive for malaria via microscopy in 2018–19 6. The most significant risk factors were considered in this study that is; indoor residual spraying, mosquito bed nets, wealth index, place of residence, and mother's education level, among others^7^–^10^. A lot of investment has been made in ITNs, LLINs, and IRS as malaria innovations^11^. The use of these innovations has been reported to reduce under-five malaria risk^12^–^16^. As an example, in 2019, insecticide-treated nets (ITNs/LLINs) protected an estimated 46% of all people at risk of malaria in Africa^2^. Specifically, in Gulu District of Uganda, IRS effectively reduced the annual prevalence of malaria from 71.5% in 2009 to 29% in 2014^9^. The WHO recommendation of vector control (ITNs/LLINs and IRs) as malaria prevention methods^2^,^17^ further informed their choice as treatment groups during analysis in this study. Estimation of average treatment effects is an important goal in evaluation research and programs^18^. Randomized control trials (RCTs) are viewed as the ideal evaluation technique for estimating treatment effects. Often, however, randomization is not feasible or permissible^19^. Matching methods can substitute RCTs in observational data^20^ as they reduce bias in these data^21^. Nearest neighbour estimators match each treated unit to a fixed number of untreated units with similar values for the pretreatment variables^18^. In this study, we assessed the effect of ITNs/LLINs on malaria risk, the effect of IRS on malaria risk, and the effect of ITNs/LLINs on IRS, using data drawn from the Uganda Malaria Indicator Survey of 2018-19.

## Methods

### Data Source and Study Population

This study used secondary data from the most recent Uganda Malaria Indicator Survey (UMIS) of 2018-19. These data were based on a two-stage cluster and stratified sampling technique qualifying them as observational data based on a complex survey design. The first sampling stage involved selecting clusters from sampling frames. Overall, 320 clusters were selected of which 84 were in urban areas and 236 in rural areas. The second sampling stage involved a systematic selection of households and a total sample size of 8,878 households was considered. The study population consists of children less than 5 years of age who were tested for anaemia and malaria infection. Blood samples for biomarker testing were collected by finger-or heel-prick from children aged 0-59 months. Each field team included two health technicians who carried out the anaemia and malaria testing and prepared the blood smears. Three questionnaires were used during the survey (household questionnaire, woman's questionnaire, and biomarker questionnaire). Basic information was collected using the household questionnaire on the characteristics of each person in the household, for example, their age and sex. The data on the age and sex of household members obtained from the household questionnaire were useful in identifying women to be interviewed for under-five children eligible for anaemia and malaria testing. The household questionnaire also captured information on the household's dwelling unit characteristics such as ownership of mosquito nets and indoor residual spraying. The results of the anaemia and malaria testing of under-five children were recorded in the biomarker questionnaire^6^. Notably, the outcome variable is malaria test result (positive and negative) which signifies malaria risk.

### Nearest Neighbour Matching (NNM)

Nearest neighbour and propensity score matching methods are the most commonly used techniques to estimate treatment response using observational data^20^,^22^. Despite the popularity of propensity score matching in observational studies, it has a major drawback of pairing individuals of the case and control in the compressed one-dimensional space of propensity scores^23^ and hence, nearest neighbour matched analysis was used instead, in this study. During analysis, the four steps recommended^24^ were followed while matching, with the first three representing the design and the fourth the analysis. These are: 1) defining closeness – the distance measure used to determine whether an individual is a good match for another; 2) implementing a matching method, given that measure of closeness; 3) assessing the quality of the resulting matched samples and 4) analysis of the outcome and estimation of the treatment effect, given the matching done in Step (3). The k:1 nearest neighbour matching form was used instead of the 1:1 form, which is the simplest NNM as the latter form can discard a large number of observations and thus would most likely lead to reduced power^24^. The Mahalanobis distance metric was applied to improve performance while matching. The nearest neighbour matching analysis estimates the average treatment effect (ATE) and average treatment effect on the treated (ATET) from observational data. The average treatment effect on the treated was estimated and used since the study intended to evaluate the treatment effects on children for whom policy or programs are intended for instead of average treatment effects on the whole population, which is useful to evaluate the expected effect on the outcome if children in the population were randomly assigned to treatment. During analysis, children in the treated and control groups were matched by taking each treated child and searching for the control child with the nearest neighbour without replacement. The ATET of interest was then obtained by averaging the difference between the outcome of the treated children and the outcome of the matched control children^25^–^27^ using the formula for the nearest neighbour matching estimator below;


$$ATET=\frac{1}{N^{T}}\sum_{i:W_{i}=1}\left[Y_{i}^{obs}-\sum_{j\in C{\left(i\right)}_{M}}w_{ij}Y_{j}^{obs}\right]=\left[\frac{1}{N^{T}}\sum_{i:W_{i}=1}Y_{i}^{obs}-\frac{1}{N^{T}}\sum_{j\in C{\left(i\right)}_{M}}w_{j}Y_{j}^{obs}\right]$$

Where *N^T^* is the number of children in the treated group (those who used ITN/LLIN or whose households used IRS), $N_{i}^{C}$ is the number of children in the control group matched with the treated child i, $w_{{-}_{ij}}$ is equal to $\frac{1}{N_{i}^{C}}$ if j is a control units of i, and zero otherwise and *w_j_* = Σ*_i_ w_ij_*. Box plots of matching over treatment levels were used to check balance of matched data. Matching was considered balanced (of similar distribution) if the matched-sample box plots were the same over the treatment levels.

## Results

### Characteristics of under-five children

A sample of 7,632 children under the age of 5 years formed the sample of this study. The children were evenly distributed by sex and anaemia status. Most of the children were from households with low wealth index (57.5%), resided in rural areas (79.2%), and were located in the western region (39.5%). The majority of the children had used an ITN or LLIN (87.4%) and resided in households that had not used IRS in the past 12 months of the survey (87.0%). The rest of the results are presented in Table 1.

**Table 1:** Distribution of Under-Five Children by Selected Background Characteristics

Background Characteristics	Category	Count	Percent
Child's sex (n=7,632)	Male	3,870	50.7
	Female	3,762	49.3
Child's age (n=7,632)	<1	1,502	19.7
	1	1,441	18.9
	2	1,502	19.7
	3	1,608	21.1
	4	1,579	20.7
Anaemia (n=7,632)	Not anaemic	3,692	48.4
	Anaemic	3,940	51.6
Household wealth index (n=7,632)	Low	4,385	57.5
	Middle	1,229	16.1
	High	2,018	26.4
Used IRS (7,592)	No	6,603	87.0
	Yes	989	13.0
Mother's education (n=6,358)	No education	1,356	21.3
	Primary	3,647	57.4
	Secondary or	1,355	21.3
	higher		
Used an ITN/LLIN (n=7,632)	No	964	12.6
	Yes	6,668	87.4
Residence (n=6,971)	Rural	5,518	79.2
	Urban	1,453	20.8
Region (n=7,632)	Central	1,189	15.6
	East	1,729	22.7
	North	1,701	22.3
	West	3,013	39.5

*Source: UMIS Data 2018-19*

### Effect of ITNs/LLINs and IRS on malaria risk

After nearest neighbour matching, the probability of malaria was 5% [ATET=-0.05; 95% CI= -0.07 – -0.02] lower in children who used ITNs/LLINs compared to the same children who did not. This indicates that sleeping under a mosquito bed net reduces the chances of malaria infection in under-five by 5% compared to not sleeping under the bed nets. The probability of malaria infection among children whose households had sprayed their dwellings in the past 12 months of the survey was 12% [ATET=-0.12; 95% CI= -0.15 – -0.09] lower compared to similar children whose households had not sprayed their dwellings in the same period. Similarly, this implies that use of IRS reduces the chances of malaria infection compared to not using it. It also indicates that IRS has a higher effect compared to use of ITNs/LLINs in reducing the probability of malaria infection in the under-five. The results further revealed that the probability of IRS is 3% [ATET=0.03; 95% CI= 0.01 – 0.05] higher among children who slept under an ITN/LLIN compared to similar children who did not sleep under these bed nets. This indicates that using ITNs/LLINs increases the likelihood of using IRS by 3% compared to not using it as a vector control measure. Results are presented in Table 2.

**Table 2:** Average Treatment Effect on the Treated (ATET) of ITN/LLIN and 1RS on malaria isk and ITN/LLIN on IRS

	Estimate	Standard error (SE)	(95% CI)
Effect of ITN/LLIN and IRS on malaria risk			
Has ITN/LLIN	-0.05	0.01	[-0.07, -0.02]^a^
Used IRS	-0.12	0.02	[-0.15, -0.09]^a^
Effect of ITN/LLIN on IRS			
Has ITN/LLIN	0.03	0.01	[0.01, 0.05]^b^

*Source: UMIS Data 2018-19. Note: Robust SE were used*

a *P < 0.001*

b *P < 0.05*

**Figure 1:**
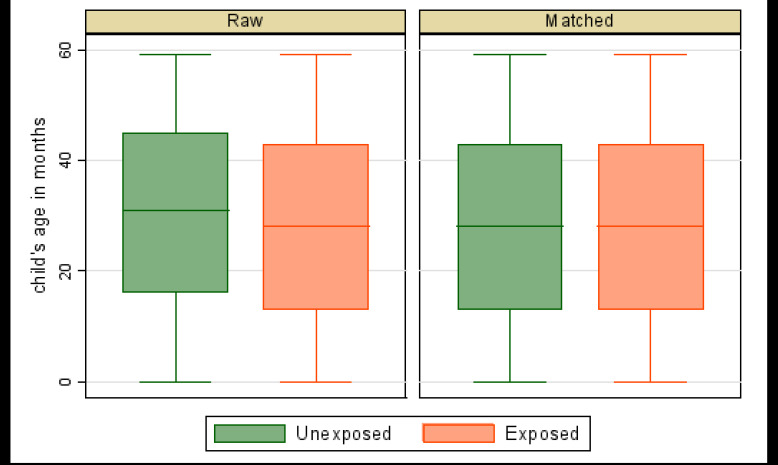
Balance plot of risk of malaria by LLIN/ITN

**Figure 2:**
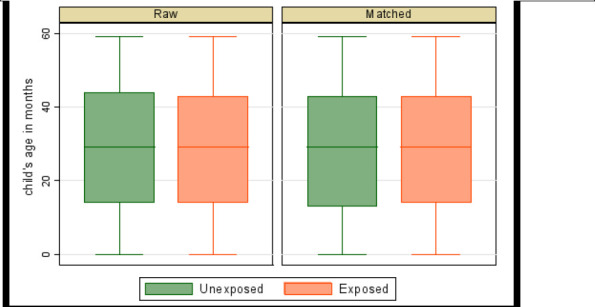
Balance plot of risk of malaria by IRS

**Figure 3:**
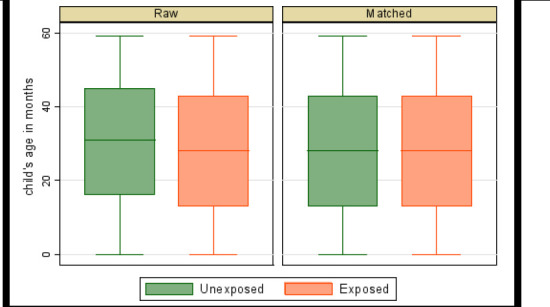
Balance plot of IRS by ITN/LLIN

The box plots (balance plots) for the matched sample are very similar across (Figures 1, 2, 3). The medians, the 25th percentiles, and the 75th percentiles appear to be the same, as well as the tails. Matching appears to have balanced matching variables (child's age in months, child's anaemia status, age of household head, sex of household head, and residence) with the child's age as the bias-adjustment covariate. Mahalanobis distance based on the child's age was used to find matches. The balance of matched data is further indicated in Table 3.

**Table 3:** Covariate balance summary of ITN/LLIN and IRS on malaria risk and ITN/LLIN on IRS

Number of observations	Effect of ITN/LLIN on malaria risk		Effect of IRS on malaria risk		Effect of ITN/LLIN on IRS	

	Raw	Matched	Raw	Matched	Raw	Matched
Total	6,931	8,406	6,931	1,974	6,931	8,406
Treated	4,203	4,203	987	987	4,203	4,203
Control	2,728	4,203	5,944	987	2,728	4,203

*Source: UMIS Data 2018-19.*

## Discussion

Findings from this study are similar to other studies^28^–^30^ as IRS was also associated with a lower risk of malaria. Similarly, ITNs/LLINs (insecticide-treated bed nets) were an important intervention for reducing malaria risk^30^–^33^. However, the effect of ITNs/LLINs was lower than that of IRS^34^. Reasons for this lower effect include; being disliked and hence misused^35^, there are species-specific differences in biting hours^36^, there is fear of chemicals, and inherent cultural beliefs^37^, among others. Results from this study further show that there was an association between ITN/LLIN and IRS; a household where IRS was done in the past 12 months was more likely to sleep under an ITN/LLIN. This is similar to a study^38^ which showed that a child under five years of age who lived in a house that had been recently sprayed was 3.1 percentage points more likely to sleep under a treated bed net.

One of the limitations of this study was the data collection procedure of one of the vector control methods. Information on whether a household used IRS was obtained by asking a household member whether the household had been sprayed against mosquitoes in the last 12 months. This was subject to recall bias as it is possible that the household member who answered the question did not remember this event or was not present at the time of spraying, which could have resulted in misreporting of IRS.

## Conclusions and Recommendations

The findings from this study illustrate a significant and negative relationship between malaria risk and ITN/LLIN use, malaria risk, and IRS during the past 12 months of the survey, but a significant positive relationship between IRS and ITN/LLIN use among the under-five in Uganda. The implementation of policies and programs towards effective use of ITN/LLIN and IRS can reduce the burden of under-five malaria in Uganda. The nearest neighbour matched analysis technique was effective in determining treatment effects. Analysts can use the matching method to determine treatment effects of malaria interventions while dealing with observational malaria data.
